# Primary Hyperhidrosis in Children—A Retrospective Study and a Short Review

**DOI:** 10.3390/life14050645

**Published:** 2024-05-19

**Authors:** Florentina Nastase, Madalina Codruta Verenca, Elena Niculet, Diana Sabina Radaschin, Camelia Busila, Claudiu Ionut Vasile, Alin Laurentiu Tatu

**Affiliations:** 1 Department of Neuropsychomotor Rehabilitation, Sf. Ioan Clinical Hospital for Children, 800487 Galati, Romania; florentina34ro@yahoo.com (F.N.); madverrec@gmail.com (M.C.V.); 2 Department of Morphological and Functional Sciences, Faculty of Medicine and Pharmacy, “Dunarea de Jos” University of Medicine and Pharmacy, 800008 Galati, Romania; helena_badiu@yahoo.com; 3 Multidisciplinary Integrated Center of Dermatological Interface Research MIC-DIR (Centrul Integrat Multidisciplinar de Cercetare de Interfata Dermatologica—CIM-CID), Dunărea de Jos University, 800201 Galati, Romania; diana.radaschin@ugal.ro (D.S.R.); dralin_tatu@yahoo.com (A.L.T.); 4 Clinical Medical Department, Faculty of Medicine and Pharmacy, Dunarea de Jos University of Medicine and Pharmacy, 800008 Galati, Romania; 5 Dermatology Department, Sfanta Cuvioasa Parascheva Hospital of Infectious Diseases, 800179 Galati, Romania; 6 “Sf. Ioan” Emergency Clinical Paediatric Hospital, Str Gheorghe Asachi nr 2, 800487 Galati, Romania; 7 Department of Psychiatry, Elisabeta Doamna Psychiatric Hospital, 800179 Galati, Romania

**Keywords:** hyperhidrosis, sweat, iontophoresis, children

## Abstract

Primary hyperhidrosis (PH) is a relatively common chronic disorder, characterized by significant and uncontrollable sweating. The predominant areas of occurrence are hands, feet, head and armpits, and it affects both men and women equally, with a false impression of increased prevalence in women. This study aims to determine the incidence of cases of hyperhidrosis, the gender of the patients and the environment of origin and to identify the most affected age groups and the distribution of hyperhidrosis, as well as creating a curve of cases within the time interval studied and their comparison with those in the specialized literature.

## 1. Introduction

Hyperhidrosis (HH) is a chronic disorder characterized by significant, unexpected and uncontrollable, bilateral and relatively symmetric sweating beyond the physiological temperature homeostatic requirements, in response to emotional stimuli or psychological stressors [1,2,3]. Although it can occur at any age, the usual onset appear between 14 and 25 years old [4,5], but the affected region is also taken into account: palmar and/or plantar in prepubertal age, axillary during adolescence [4,6]. It predominantly appears on the hands, feet, head, armpits, and in the inguinal area, and can affect one or more region of the body [7,8]. There is a false impression of increased prevalence in women due to the fact that women are more likely report and seek treatment, but hyperhidrosis affects both men and women equally [7,9,10]. There is probably a genetic factor involved in hyperhidrosis, because 30–65% of patients describe a positive family history. It may have partial penetrance, variable phenotype and an autosomal dominant transmission [4,11,12]. Loci for hyperhidrosis have been mapped on 14q11.2-q13 and 2q31.1 [4,13,14,15].

The cause of primary hyperhidrosis is unknown or not well understood. The most likely etiology is a neurogenic overactivity or a hyperexcitability of the eccrine gland circuits [16]. Individuals with PH have an increased response to stress and a higher basal level of sweating [17,18].

It is physiologically important and vital to sweat. There are two types of sweating: thermoregulatory (important to maintain the homeostasis and the body temperature) and emotional. The eccrine glands constitute most of the sweat glands, distributed all over the body and they produce a hypotonic to plasma thin secretion. The highest density is in the palms, soles and armpits [19]. Soon after birth, normal sweating of the soles and palms begins, while axillary sweating begins at puberty. This is in relation to the development of apocrine glands [20]. There are no changes in the morphology of the sweat glands. The parasympathetic and sympathetic nervous system dysfunction in hyperhidrosis is complex [19,21].

## 2. Materials and Methods

The study was initiated after the approval of the Medical Council of the Sf. Ioan Emergency Clinical Hospital for Children, Galati, Romania, no. 31117/04.12.2023.

This is a retrospective study conducted in the Department of Neuropsychomotor Rehabilitation of the hospital between January 2014 and December 2023. We analyzed the electronic database, the consultation and the treatment files of all the children who presented during this period. The criteria to be included were under 18 years old at the time of consultation and diagnosed with hyperhidrosis. The exclusion criteria included patients with contraindications to performing iontophoresis: epilepsy, skin lesions or infection.

The primary objective for this study was to determinate the incidence of cases with the above diagnosis, the gender of the patients and the environment of origin. Other objectives were to identify the most affected age groups and the distribution of hyperhidrosis, as well as elaborating a curve of cases within the time interval studied and their comparison with those in the specialized literature.

## 3. Results

**Figure 1:**
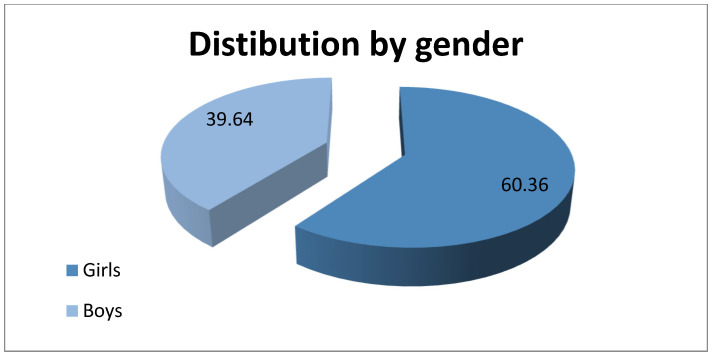
Distribution of patients by gender.

We found 111 patients who correspond to the inclusion and exclusion criteria, of whom 67 (60.36%) were girls and 44 (39.64%) were boys. Figure 1 shows the diagram of the distribution by gender. The mean age of the group was 10.59 ± 2.87, ranging from 6 to 17. The average number of diagnosed patients per year was 6.7 girls and 4.4 boys, values comparable to those in the specialized literature, this disease being more frequent among the female population compared to the male population. Among the 111 patients diagnosed with hyperhidrosis, 89 (80.18%) were from an urban environment and 22 (19.82%) from a rural environment. Considering that Galati County, the region where the study was carried out, has an urban population approximately equal to the rural one, 259,071 and 237,821, respectively, we can conclude that the significant difference between the number of patients from the urban environment compared to the rural one is due to addressability and easier access to the medical services of those in the urban environment. The average number of patients diagnosed per year was 8.90 from the urban and 2.2 from the rural area.

**Figure 2:**
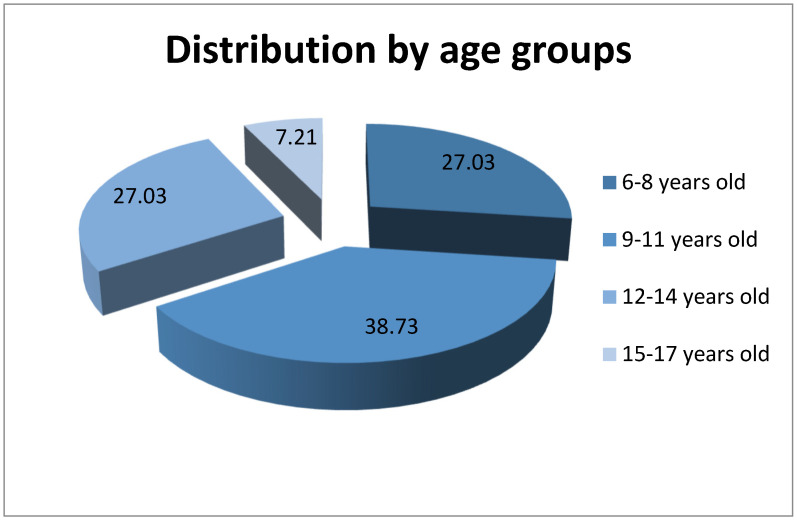
Distribution of patients by age groups.

The distribution by age groups provides the following data, represented in Figure 2:

- 6–8 years old—30 children (27.03%), 27 (90%) girls and 3 (10%) boys
- 9–11 years old—43 children (38.73%), 25 (58.14%) girls and 18 (41.86%) boys
- 12–14 years old—30 children (27.03%), 15 (50%) girls and 15 (50%) boys
- 15–17 years old—8 children (7.21%), 0 (0%) girls and 8 (100%) boys

From our observations, it appears that this condition is more common in the case of girls who come from the urban area and are aged between 6 and 11 years old.

**Figure 3:**
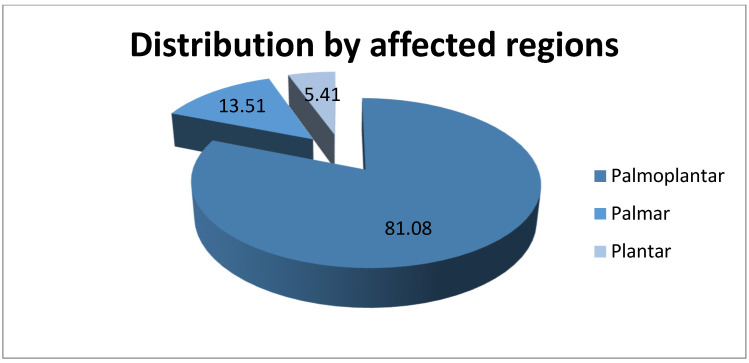
Distribution of patients by affected regions.

Palmoplantar regions were the most affected areas by hyperhidrosis, with 90 (81.08%) cases registered, followed by the palmar region with 15 (13.51%) cases and the plantar with 6 (5.41%) patients. This information is represented in Figure 3. It should be noted that the patients included in the study suffer from palmar and/or plantar hyperhidrosis because these were the areas treated by iontophoresis in our clinic. The data obtained are in accordance with the specialized literature, which shows that the palmoplantar combination is the most common. 

A family history is reported in 60 (54.10%) children, having a parent, a brother or a sister with excessive sweating, diagnosed or not with hyperhidrosis; the remaining 51 (45.90%) patients had no family history. All these data are summarized in Table 1.

**Figure 4:**
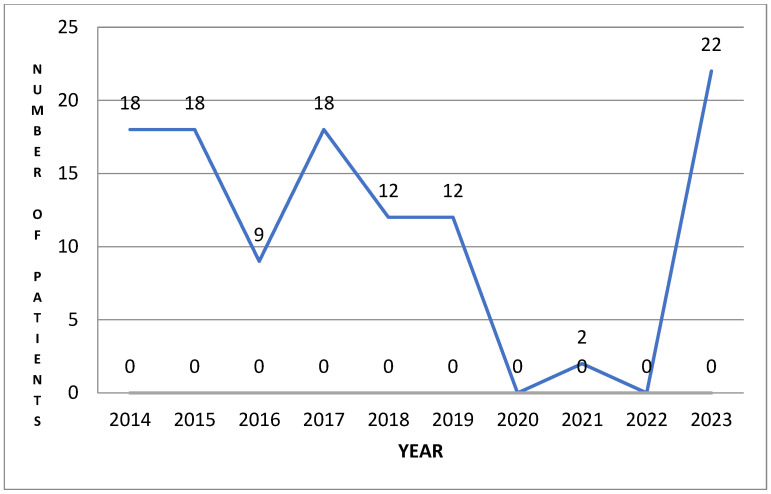
Distribution of patients/year.

Analysis of the obtained distribution curve of patients diagnosed with hyperhidrosis in the 10 years (1 January 2014–31 December 2023) and who benefited from treatment in the institution mentioned above, is presented in Figure 4 and has a roughly linear aspect from 2014 to 2019, after which it decreases suddenly to 0 during the 3 years of the pandemic, after which it returns to the initial number of patients. We note that the two patients who presented during 2021 came for other pathology—back pain and post-humeral fracture. These observations support the idea that patients do not consider hyperhidrosis an urgent problem that can be improved by treatment. 

In our clinic, we treat patients with various pathologies: cerebral palsy, genetic syndromes, vertebral static disorders, etc. A total of 63 (56.76%) patients were diagnosed by a dermatologist before presenting to us for iontophoresis and 48 (43.24%) patients presented for other pathologies, scoliosis (18), kyphosis (15), flat foot (3), genumvalgum (3) and post-trauma status or pain (9); during the consultation, a diagnosis of primary hyperhidrosis was made and they were referred to a dermatologist for confirmation and topical treatment. The treatment applied to all patients was iontophoresis, without completing a severity scale before or after. At the end of the 10 days of treatment, 82 (73.87%) patients stated that they sweated less, while 29 (26.13%) patients declared that the sweating was the same as before the treatment.

## 4. Discussion

Hyperhidrosis or excessive sweating is a pathologic condition characterized by sweating beyond that necessary for thermoregulation [22]. HH can be classified in:

- Primary, idiopathic—bilateral, focal and symmetric distribution, with one or more areas affected, which occurs in healthy individuals [4,8,16,23]. The overproduction of sweat is episodic, triggered by stress and emotions, occurs during the day and disappears during sleep [24].
- Secondary—after medication consumption or from an underlying medical condition, characterized by generalized exaggerated sweating [23]. This usually affects older persons, is asymmetric, may occur at night, during sleep, and is not associated with a familial history [24,25]. The most common triggers are cardiac diseases, endocrinopathies, proliferative diseases, metabolic and psychiatric disorders, nervous system diseases [24,26], or as an adverse reaction to drugs: Cliclooxygenase inhibitors, opioid analgesics, antiviral medication, antibiotics or hypotensive medicaments [24,27].

Before making the diagnosis of primary hyperhidrosis, secondary hyperhidrosis is excluded [4,16,23]. A comprehensive medical history and physical examination must be taken, which presents all information for differentiating the two types of hyperhidrosis [4,28], because PH has distinctive features, enabling its diagnosis to be made exclusively by physical examination and medical history [23,29]. The features are healthy and young with or without a family history; focal, bilateral and symmetric involvement of axillae and/or hands and/or feet; aggravating emotional, thermal and/or physical stimuli; cessation of symptoms during sleep/night [4]. The following diagnostic criteria were proposed by the Multi-Specialty Working Group on Hyperhidrosis:

- 6 or more months of focal, detectable, visible, exaggerated sweating without an explanation.
- 2 or more of the following features:
  ○ at least one episode/week
  ○ bilateral and symmetrical sweating
  ○ debut before 25 years old
  ○ family history of hyperhidrosis
  ○ absence of focal sweating during sleep
  ○ interference with daily activities [3,4,7,18,29].

The sweating mechanism is important for the body’s thermoregulation. Hyperactivity of the sweat glands can appear in certain physiological conditions: obese people, during and after exercise and in menopause [7].

Hyperhidrosis, like other diseases, is not very common, therefore it requires a special team and careful treatment of the symptoms in order to make the correct diagnosis.

Another example of an extremely rare condition is nasopharyngeal tuberculosis. Nasopharyngeal tuberculosis is rare, representing around 1% of all upper air-way localizations and the most common presentation is in the form of adenoids. Tuberculous glossitis (oral tuberculosis) is even scarcer and may present in various clinical forms, usually mimicking a malignant neoplasm, or, less often, trauma or other infectious lesions. Oropharynx tuberculosis is usually misdiagnosed as hypertrophic chronic tonsillitis [30].

After determining the diagnosis of primary hyperhidrosis, the severity is established [4,18]. As the sweating is episodic, it is impossible to observe during the clinical examination. When it is visible, the severity can be determined based on the extent of sweat stains on clothes, useful for axillary area: diameter < 5 cm—normal; diameter 5–10 cm—mild hyperhidrosis; diameter 10–20 cm—moderate hyperhidrosis; diameter > 20 cm—severe hyperhidrosis [4,31]. For hands, the following classifications are available: mild—sweaty hands, without droplets; moderate—sweat to the tips of the finger; severe—sweat drips off [31]. An accepted sweat quantity for hyperhidrosis diagnosis does not exist; in studies, the normal value appears as <1 mL/m^2^/min [4,32]. Axillary hyperhidrosis is diagnosed at sweat values over 100 mg/5 min/axillae for men and 50 mg/5 min/axillae for women [4,32,33,34]. Palmar hyperhidrosis is diagnosed at sweat values over 30–40 mg/min [4,35,36]. 

The measurement tools available for quantitative sweat production are the starch-iodine (Minor’s) test and gravimetry test [29]. Gravimetry consists in measuring the amount of perspiration produced in one area in a certain period of time [33,34]. After drying the affected area, a pre-weighed gauze is placed on the region and then the difference between the two weights is determined, expressed in mg/min. The limitation of this test is that it does not take into account the size of the sweating area [4]. The Minor starch-iodine test is an inexpensive and simple test for identifying the affected area, recognizing the presence and assessing the severity of hypersecretion [4,23,37,38]. This test is used to identify areas with different perspiration intensities, not to quantify hyperhidrosis severity [4]. With this test, the intensity of sweating can be classified using the 6-grade Intensity Visual Scale and the final purple color from Minor’s test, as follows:

- 0—no sweating
- 1—initial
- 2—mild
- 3—moderate
- 4—intense
- 5—excess sweating [4,39].

Less quantitative tests for hyperhidrosis measurements are the ninhydrin test, based on the chemical reaction between amino-acids from sweat and ninhydrin, dynamic quantitative sudometry, and evaporimetry [4].

Daily life is also affected by excessive sweating. For example, simple tasks like homework or writing important documents can be ruined by sweat, and the paper can be smeared and stained with ink by dripping sweat. The impact is negative, especially on self-esteem, emotional state, occupational productivity or interpersonal relationship, including avoiding shaking hands. It is difficult to grip tools with sweaty hands, to use electronic devices or to play musical instruments. Patients with palmar hyperhidrosis reported frequent electric shock, dropping glass objects or difficulty in writing or drawing. Professional issues for those with axillary hyperhidrosis include the need to change clothes or anxiety in presenting to other people because of stained clothes and the resulting embarrassment [22]. The World Health Organization defines quality of life (QoL) as an individual’s own perception of QoL compared with expectations. This can be influenced by culture, values, social standards and by goals. The literature describes numerous validated tools for assessing the quality of life of patients with dermatological problems. They are useful for evaluating treatment outcomes and for determining the extent of symptoms. It also helps raise awareness of the disease by identifying patients with increased medical care needs and provides insight into patients’ lives [1]. 

In addition to the discomfort created, the complications of hyperhidrosis can be various dermatological conditions: skin infection, due to humidity; dyshidrotic eczema, i.e., pompholyx, in palmar hyperhidrosis; unpleasant odor, tinea pedis, skin maceration and onychomycosis in plantar hyperhidrosis [40]. Another complication of hyperhidrosis is bromhidrosis, a chronic condition characterized by an unpleasant smell [41], which often occurs in an axillary manner. Commercial antiperspirants and antibacterial soaps can be useful to control it, in addition with hair removal, changing underwear, frequent baths and topical applications of aluminum salts [40].

The treatment of patients with hyperhidrosis is not simple as in the case of some other medical conditions. For example, in the case of oral mucositis, numerous studies have highlighted the benefits of honey for oral mucositis. Through its analgesic, anti-inflammatory, anti-cancerous and antibacterial action, honey has proved to have a major impact on the patient’s quality of life and nutritional status by promoting tissue epithelialization and healing of the chemoradiotherapy-induced lesions [42]. 

The goal is to reduce the sweating volume to an acceptable level for the patient. First of all, before developing a treatment plan, it is important to understand the expectations and motivation of the patient and to warn about limitations and complications of the treatment [43]. The National Institute for Health and Care Excellence (NICE) provides recommendations for the initial treatment of hyperhidrosis: avoiding alcohol, spicy foods, stress, emotional triggers and wearing non-occlusive footwear or clothes [20]. 

The first choice in the treatment of hyperhidrosis is topical applications. The result of the agents applied to the skin occurs through their astringent effect on the epithelium and the sweat gland or by blocking the ducts of the eccrine glands [36,43]. Topical anticholinergics have proved effectiveness in the reduction of symptom severity in 70% of cases for up to 4 weeks. The administration methods are aerosol, contact sensitization and iontophoresis [3].

Aluminum chloride, a metallic salt, is the most widely used antiperspirant to control the mild and moderate axillae, and palms and soles hyperhidrosis [3,7,24,43]. Aluminum ions are absorbed by cells lining the sweat ducts, damaging the epithelial cells and forming a plug that blocks the excretion of sweat [3,8,17,24]. Because the secretion of the sweat glands continues, miliaria can appear due to accumulation of sweat [22,40]. The effect is not permanent because the epithelium is renewed and, with this process, the function of the sweat glands also returns. Reapplication is necessary daily until the desired response is achieved, then once or twice a week [3,44,45]. For the treatment of severe forms of hyperhidrosis when sweat reacts with aluminum and forms an irritating acid, i.e., hydrochloric acid, anhydrous ethyl alcohol is added [8,36]. The use of this product requires proper knowledge to decrease irritation and improve efficacy [17]. The application must be to dry skin, before sleep, kept for 6–8 h and washed off afterwards. Effectiveness can be increased by covering the axillae with a T-shirt, the scalp with a shower cap, the palms with gloves, and feet with socks [8]. The most common adverse effects of using this topic are skin irritation and redness [17,20,24,46], which may lead to discontinuation or nonadherence to treatment [36]. This topical treatment also has disadvantages, such as amelioration of symptoms only for a short period of time, ineffective in severe forms [8], and being time-consuming and messy [43]. Concerns about influence of persistent exposure to aluminum from antiperspirants on a higher prevalence of breast cancer and Alzheimer’s disease have not been confirmed in studies [24]. Topical preparations of aluminum chloride are also popular in children’s hyperhidrosis due to the ease of application and their safety [47].

Systemic treatment is reserved for patients who do not respond to topical treatment. Systemic anticholinergic agents are the first choice, but adverse reactions force one third of patients to stop the treatment [2,24]. Glycopyrrolate is an oral drug widely prescribed, which has no central nervous system side effects because it does not penetrate the hemato-encephalic barrier, and therefore the dose can be increased until the desired result is achieved [24,48,49]. Anticholinergic agents are contraindicated in pyloric stenosis, myasthenia gravis, paralytic ileus, bladder outlet obstruction or closed-angle glaucoma [24].

Electric current has been used to introduce ions into the skin since the 1930s, a process called iontophoresis [8,47]. It is defined as the introduction through intact skin of an ionized substance [43]. The areas treated by this procedure are the hands and feet, with good results, but the armpits represent a difficult area to apply due to their anatomy [40,47]. Although it has been used for many years, the mechanism of action is unknown, but theories include blockade of sympathetic nerve transmission, the accumulation of hydrogen ions decreasing pH, and ion depositions clog the eccrine sweat glands [17]. This type of treatment requires repetition of a variable number of applications to achieve the desired result, and then the whole process is repeated after a few months, from 2 to 14 months [43]. There are two types of applications: with the feet or hands immersed in water and with moist electrodes applied on the skin. It is time consuming, but the results are effective in 81% of cases [19]. Iontophoresis is a safe procedure, but sometimes discomfort can occur as a tickling or burning sensation [22]. This treatment has mild complications, such as rash, erythema—mild and completely transient, treatable with moisturizers or topical corticosteroids—and transient paresthesia [43]. No cases of compensatory sweating have been reported after this treatment [22]. The contraindications are pregnancy, epilepsy, patients with pacemaker or metal implants [24].

The botulin toxin is odorless, colorless and tasteless and is a neurotoxin zinc-dependent endo-protease, the most poisonous known substance [8]. It is produced by gram-positive bacillus Clostridium botulinum, which is able to block neurotransmission [43]. In 1946 it was isolated in crystalline form and, after 4 years, it was discovered that it can paralyze a hyperactive muscle. Patients can develop antibodies against it, but it is extremely rare because the dose used to treat hyperhidrosis is very small [8]. It is an alternative to iontophoresis for patients with axillary hyperhidrosis. Botulin A toxin is the most widely used in dermal–subcutaneous injections in the affected areas and acts by temporarily inhibiting the release of acetylcholine [20]. Since the procedure is painful, analgesics can be used: oral sedation medication, nerve blocks, topical lidocaine cream, intravenous regional anesthesia. Approximately 20 injections distributed in the area delimited by the minor starch-iodine test are necessary. After 2–4 days, there is a noticeable reduction in sweating and after 2 weeks this should be substantial. The sweat function returns gradually after an average period of 7 months. This treatment is repeated every 4–17 months. The disadvantages are the multiple uncomfortable injections, repeated after a period of time, the need to use an analgesic or anesthetic and the risk of spread of the toxin into the intrinsic muscle of the hand [8]. The most common adverse effects are pain, localized hemorrhage, indigestion [50], or antibody formation (extremely rare, but possible), which lead to a reduction in the effectiveness of the treatment, or other possible adverse reactions or situations [42,51,52,53,54]. The contraindications to Botulin A toxin are hypersensitivity to albumin, myasthenia gravis, or Eaton–Lambert syndrome [36]. Botulin B toxin has a shorter duration, a faster action and more side effects than Botulin A toxin [36,55].

Surgical therapy is reserved as a last line treatment, after the failure of other less invasive options [56]. It is a valuable choice, but it should be reserved for the more aggressive forms due to the potential complications [43]. The technique used is to remove or damage the sweat glands [56]. Radical excision of skin rich in glands is performed under local or general anesthesia. The advantage of this procedure is the unique operation with a permanent effect on the reduction of hyperhidrosis, but it also presents many disadvantages in comparison to other local surgical therapy: skin necrosis, keloids, hypertrophic scars and long convalescence. Another option for axillary hyperhidrosis is subcutaneous curettage with a spoon-shaped currette or a sharp gynecological currette, performed under local or general anesthesia. Apart from the advantage of a small scar without tension, the disadvantages are hematomas, skin necrosis, delayed wound healing or the possibility of recurrence due to incomplete removal [10].

Another surgical option for treating hyperhidrosis is endoscopic thoraco-sympathectomy (ETS), reserved as the last step in severe cases after failure of other less invasive options [20]. The procedure consists in removing the Th2/3 sympathetic ganglia and the improvement is long-term in 79% of cases [56]. The effect of interruption of the sympathetic chain is permanent ceasing of sweating in the distribution area of the nerve. In the case of treatment of palmar hyperhidrosis, only the dominant hand can be treated, thus reducing the risk of developing compensatory sweating [43]. The main adverse effect is the development of compensatory hyperhidrosis—irreversible and possibly worse than the original, located in the chest area, abdomen, legs or back [56]. Other postoperative complications are haemothorax, pneumothorax, dry hands, gustatory sweating, altered taste, Horner’s syndrome, and recurrence of symptoms [3]. Contraindications for ETS are chest scars or pulmonary disease [40].

Another therapeutic option in HH is laser technology, used externally to destroy the glandular tissue by subdermal coagulation. The side effects are limitation of mobility and transient pain for 1–4 weeks after procedure. Microwave or ultrasound devices can also be used [40].

Hyperhidrosis is a condition with a significant emotional and psychological impact on the patient’s social life and his family. The numerous therapeutic options prove that there is an interest in controlling and treating this unpleasant and problematic disease. Most current therapies are reversible, being therefore only temporary solutions [40].

## 5. Conclusions

1. Hyperhidrosis has a significant impact on the patient. The therapeutic options are varied, each having advantages, disadvantages and adverse effects.
2. Hyperhidrosis is underdiagnosed and many cases are not reported and treated, due to the lack of knowledge of therapeutic options for pediatric patients.
3. Children are more prone to emotional suffering; their social and psychological development being affected.
4. The increased prevalence in girls is false, because they are more likely to report and seek treatment.
5. In this study, an increased ratio of girls/boys is observed in the age range between 6 and 11 years, after which the ratio equalizes at 12–14 years, and then reverses in the 15–17 age group.
6. Palmoplantar combination is the most frequent approach, followed by palmar and then plantar.
7. The involvement of the genetic factor is supported in this study by the occurrence of excessive sweating in a family member in 54.10% of cases.
8. Due to the approximately equal numbers among the urban and rural populations of Galati County, we can conclude that the significant difference between the number of patients from urban and rural areas, 89 and 22, respectively, is due to the easier access to medical services and therapeutic options for urban patients.
9. An aspect that supports lack of knowledge of this disease, of therapeutic possibilities and of the important negative consequences on quality of life and the psyche of children discovered in this study is the drop to 0 in addressability during the COVID-19 pandemic, a period when priorities were different.
10. Iontophoresis is effective in improving symptoms, and we will carry out future studies to highlight the improvement in quality of life based on valid questionnaires.
11. Effective treatment, whether topical, systemic or invasive, should aim for optimization of patients’ quality of life.
12. Before proceeding to permanent and possible irreversible surgical or invasive treatment, conservative therapies should be tried.
13. Hyperhidrosis has a profound mental and physical impact on patients, which are under-diagnosed and undertreated, and it deserves as equal consideration as other more well-known skin problems.
14. This study aims to raise the alarm about the need for awareness of this frequently underdiagnosed disease, which has serious mental and emotional consequences.

## Figures and Tables

**Table 1:** Patients’ characteristics.

	Clinical Profile	n = 111 (%)
Age		
	6–8	30 (27.03%)
	9–11	43 (38.73%)
	12–14	30 (27.03%)
	15–17	8 (7.21%)
Sex		
	Girls	67 (60.36%)
	Boys	44 (39.64%)
Environment		
	Urban	89 (80.18%)
	Rural	22 (19.82%)
Site of involvement		
	Palmoplantar	90 (81.08%)
	Palmar	15 (13.51%)
	Plantar	6 (5.14%)
Family history		
	Yes	60 (54.10%)
	No	51 (45.90%)

## Data Availability

The data presented in this study are available on request from the corresponding author.
